# ROR2 promotes invasion and chemoresistance of triple-negative breast cancer cells by activating PI3K/AKT/mTOR signaling

**DOI:** 10.32604/or.2024.045433

**Published:** 2024-06-20

**Authors:** XIA DA, HAN GE, JUNFENG SHI, CHUNHUA ZHU, GUOZHU WANG, YUAN FANG, JIN XU

**Affiliations:** 1 Department of Breast and Thyroid, Nanjing First Hospital, Nanjing Medical University, Nanjing, China; 2 Department of General Surgery, The First Affiliated Hospital of Nanjing Medical University, Nanjing, China; 3 Department of Oncology, Nanjing First Hospital, Nanjing Medical University, Nanjing, China; 4 Department of Pathology, Nanjing First Hospital, Nanjing Medical University, Nanjing, China

**Keywords:** Receptor tyrosine kinase-like orphan receptor 2, Triplet-negative breast cancer, Proliferation, Apoptosis, PI3K/AKT/mTOR signaling, Metastasis

## Abstract

**Objective:**

This study aimed to investigate the role of receptor tyrosine kinase-like orphan receptor 2 (ROR2) in triple-negative breast cancer (TNBC).

**Methods:**

ROR2 expression in primary TNBC and metastatic TNBC tissues was analyzed by immunohistochemical staining and PCR. ROR2 expression in TNBC cell lines was detected by PCR and Western blot analysis. The migration, invasion and chemosensitivity of TNBC cells with overexpression or knockdown of ROR2 were examined.

**Results:**

ROR2 expression was high in metastatic TNBC tissues. ROR2 knockdown suppressed the migration, invasion and chemoresistance of TNBC cells. ROR2 overexpression in MDA-MB-435 cells promoted the migration, invasion, and chemoresistance. Moreover, ROR2 knockdown in HC1599 and MDA-MB-435 adriamycin-resistant cells enhanced chemosensitivity to adriamycin. ROR2 could activate PI3K/AKT/mTOR signaling in TNBC cells.

**Conclusion:**

ROR2 is upregulated and promotes metastatic phenotypes of TNBC by activating PI3K/AKT/mTOR signaling.

## Introduction

Breast cancer is a common cancer in the women [1,2]. Triple-negative breast cancer (TNBC) is named due to negative expression of estrogen receptor (ER), progesterone receptor (PR) and human epidermal growth factor receptor 2 (HER2) [3,4]. Although treatment can extend the survival of patients, one in five patients with TNBC will develop chemoresistance and metastatic disease within five years after the diagnosis [5]. TNBC has the worst outcome among breast cancer subtypes, with five-year survival rate of 91%, 65%, 11% for localized, regional and metastatic TNBC, respectively [6].

Receptor tyrosine kinase-like orphan receptor 2 (ROR2) is implicated in diverse cancer types, including breast cancer [7]. ROR2 activated phosphatidylinositol-3-kinase/AKT/mammalian target of rapamycin (PI3K/AKT/mTOR) in multiple myeloma [8]. ROR2 could act as an oncogene and promote breast cancer by activating PI3K/AKT and MAPK/p38 pathways [9,10].

Notably, PI3K signaling is activated in approximately 60% TNBC patients [11]. PI3K/AKT/mTOR signaling inhibitors combined with chemotherapy lead to better survival of patients with metastatic TNBC [12].

Therefore, this study aimed to analyze the expression of ROR2 in metastatic TNBC and investigate whether ROR2 regulates PI3K/AKT/mTOR signaling in metastatic TNBC.

## Materials and Methods

### Immunohistochemistry

The study was approved by Ethics Committee of Nanjing First Hospital and all patients provided informed consent. Total 40 breast cancer specimens were used, including 20 from patients with TNBC primary cancer and 20 from patients with TNBC metastatic cancer (metastasized to the brain). The tissues were cut into 4 µm sections after fixing in formalin and embedding in paraffin. After incubation with ROR2 antibody (EnoGene), the staining was detected by DAB kit (Beyotime).

### Cell culture

Five TNBC cell lines BT-549, MDA-MB-435, Hs 578T, MDA-MB-468, and HCC1599 were obtained from American Type Culture Collection (Manassas, VA, USA) and cultured in high-glucose DMEM (Gibco) supplemented with 10% fetal bovine serum (FBS) (Gibco) at 37°C in humid incubator with 5% CO_2_. MDA-MB-435/Adr, an Adriamycin-resistant TNBC cell line (Enjing Biotech) was cultured in RPMI 1640 medium supplemented with 10% FBS. Cells were transfected with ROR2 interfering plasmid siROR2, ROR2 plasmid pLenti-ROR2 and the controls as described in our previous study [9].

### CCK-8 assay

Cell viability was examined by CCK-8 assay (EnoGene, New York, USA). Approximately 1 × 10^4^ TNBC cells were seeded in each well of 96-well plates. After culture, 10 μL of CCK-8 solution was added and incubated for 4 h, and absorbance at 450 nm was measured by microplate reader (Thermo Fisher, Waltham, MA, USA).

### Wound healing assay

Cells were seeded in 6-well plates (Corning, Corning, NY, USA). When cells reached 90% confluency, cell monolayers were scratched with tips. Wound healing was monitored at 0 and 24 h after scratch, and the images were captured under a light microscope with 10× objective. The wound area (cell-free area) was measured by using a digital camera fitted to the microscope. The wound area in control group was set as 1 to calculate relative wound area in experimental groups.

### Cell invasion assay

About 1 × 10^5^ cells were seeded in serum-free medium on the upper surface of modified Boyden chambers (Millipore, USA) and the lower chamber was loaded with 10% FBS. After incubation for 24 h, the invading cells were stained with 0.5% violet dye and counted under a light microscope with 20× objective in five randomly selected fields.

### Flow cytometry

Cell apoptosis was detected using apoptosis detection kit (Enjing, China). Briefly, the cells (5 × 10^5^) were washed and resuspended in 500 μL binding buffer. The cells were then incubated with 5 μL Annexin V-FITC and 5 μL propidium iodide on the ice in the dark. Fluorescence of 1 × 104 cells per sample was analyzed on a flow cytometer (BD Biosciences, UK).

### PCR

Total RNA was extracted using TRIzol (Life Technologies, USA), and used for cDNA synthesis with RevertAid First Strand cDNA Synthesis Kit (Thermo Scientific). PCR was performed using MasterMix (Abcam, China). The sequences of the primers (Generay Biotech, Shanghai, China) were: GAPDH 5′-CCTCTGACTTCAACAGCGACAC-3′ and 5′- CTGTTGCTGTAGCCAAATTCGT-3′ (121 bp), ROR2 5′-AGTGTCCCGGACTTCAGGT-3′ and 5′-CCTTGCAGTGCAGAATTGCC-3′ (173 bp). PCR condition was: denature at 94°C for 2 min, then 94°C for 20 s and 60°C for 20 s for 40 cycles. Each sample was tested in triplicates with GAPDH as the control.

### Western blot analysis

Cells were lysed on the ice using RIPA buffer supplemented with protease and phosphatase inhibitors. After quantitation with BCA kit (Enjing, Nanjing, China), 20 μg protein was resolved on 10% SDS-polyacrylamide gel and transferred to polyvinylidene difluoride membrane (Millipore, Bedford, MD, USA). The membrane was blocked with 5% skim milk (w/v) for 1 h and washed with TBST (Tris-buffered saline TBS-Tween 20). The membrane was incubated with primary antibodies against ROR2 (EnoGene, Cat. E38PA4208, USA), Akt (Phospho-Ser473) (EnoGene, Cat. E011054-2, USA), Akt (Ab-473) (EnoGene, Cat. E021054-2, USA), mTOR (Abcam, Cat. ab134903, UK), mTOR (phospho S2448) (Abcam, Cat. ab109268, UK), PI3Kinase p85 alpha (phospho Y607) (Abcam, Cat. ab182651, UK), PI3Kinase p85 alpha (Abcam, Cat. ab191606, UK), E-cadherin (Abcam, Cat. ab227639, UK), MMP2 (Abcam, Cat. ab181286, UK), N-cadherin (Abcam, Cat. ab76011, UK), SNAIL (Abcam, Cat. ab216347, UK), Vimentin (Abcam, Cat. ab16700, UK), and GAPDH (EnoGene, Cat. E1C604-2, USA) at 4°C overnight. The membrane was then incubated with secondary antibody (EnoGene, NY, USA) for 1 h at room temperature. The bands were detected with enhanced chemiluminescence kit (Millipore, MA, USA) and quantified with ImageJ version 1.48 (NIH, Bethesda, MD, USA).

### Statistical analysis

The data are presented as mean ± standard deviation (SD). Comparisons for multiple groups were performed by one-way analysis of variance, with significance set as *p* < 0.05.

## Results

### ROR2 expression was positively correlated with TNBC metastasis

**Figure 1:**
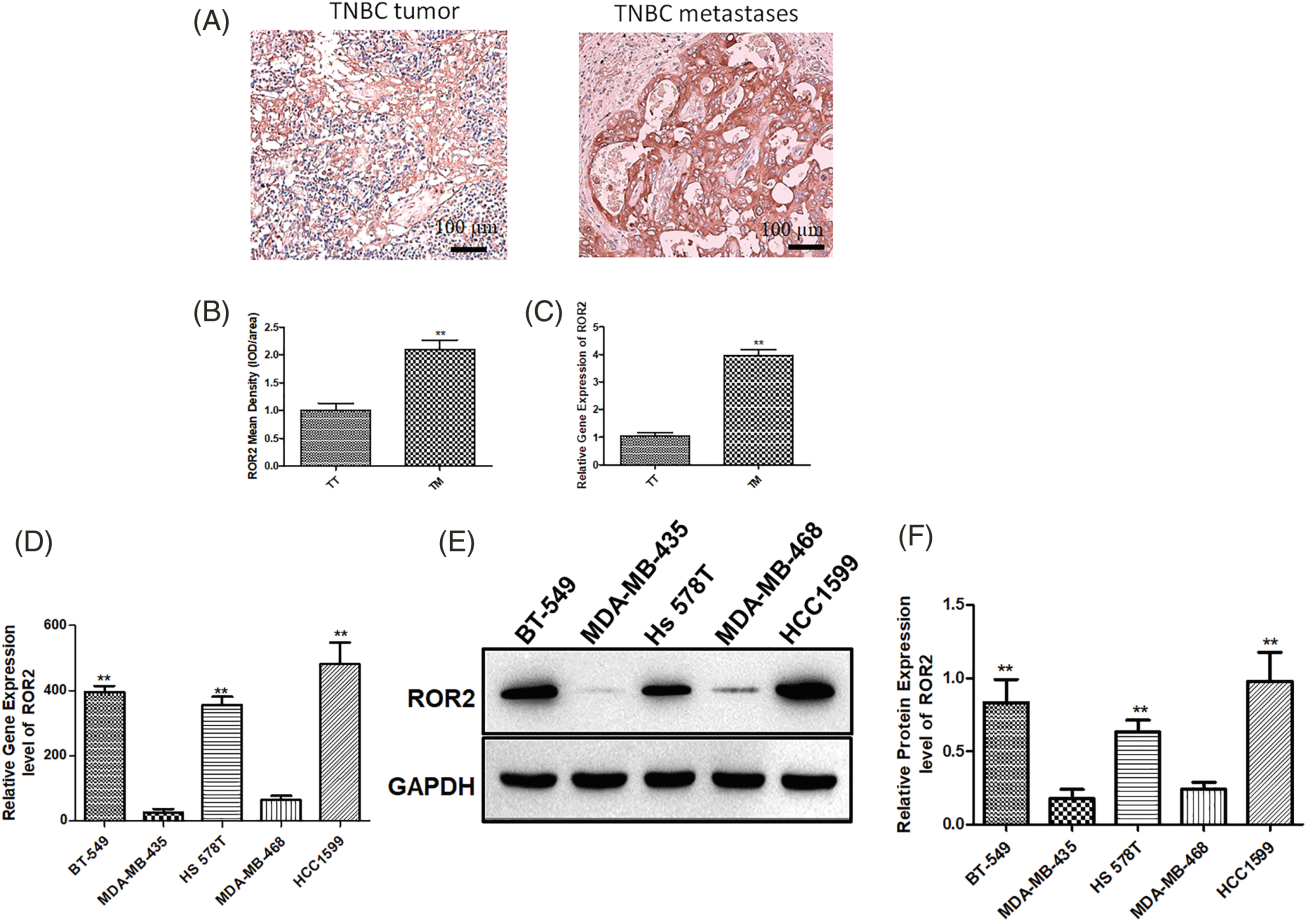
ROR2 is upregulated in TNBC cancer tissues. ROR2 expression was examined by IHC (A and B) and RT-qPCR (C) in primary TNBC and metastatic TNBC tissues (n = 20). Scale bar: 100 μm. **p < 0.01. TT: TNBC tumor, TM: TNBC metastases. (D) ROR2 expression in TNBC cell lines BT-549, MDA-MB-435, Hs 578T, MDA-MB-468 and HCC1599. (E and F) Relative protein expression of ROR2 in BT-549, MDA-MB-435, Hs 578T, MDA-MB-468 and HCC1599 cells. Data are presented as mean ± SD (n = 3, **p < 0.01 *vs*. MDA-MB-435 or MDA-MB-468 cells).

First, we examined ROR2 expression in primary and metastatic TNBC tissues by IHC staining and PCR. Strong ROR2 staining was observed in the invasive and metastatic TNBC tissues (Figs. 1A and 1B). In TNBC tumors with metastasis, ROR2 expression was higher than that in metastasis-negative tumors (Fig. 1C). Furthermore, we analyzed ROR2 mRNA and protein levels in TNBC cells. Among them, ROR2 expression was the highest in HCC1599 cells and the lowest in MDA-MB-435 cells (Figs. 1D–1F). We selected HCC1599 and MDA-MB-435 cells for ROR2 knockdown and overexpression, respectively.

**Figure 2:**
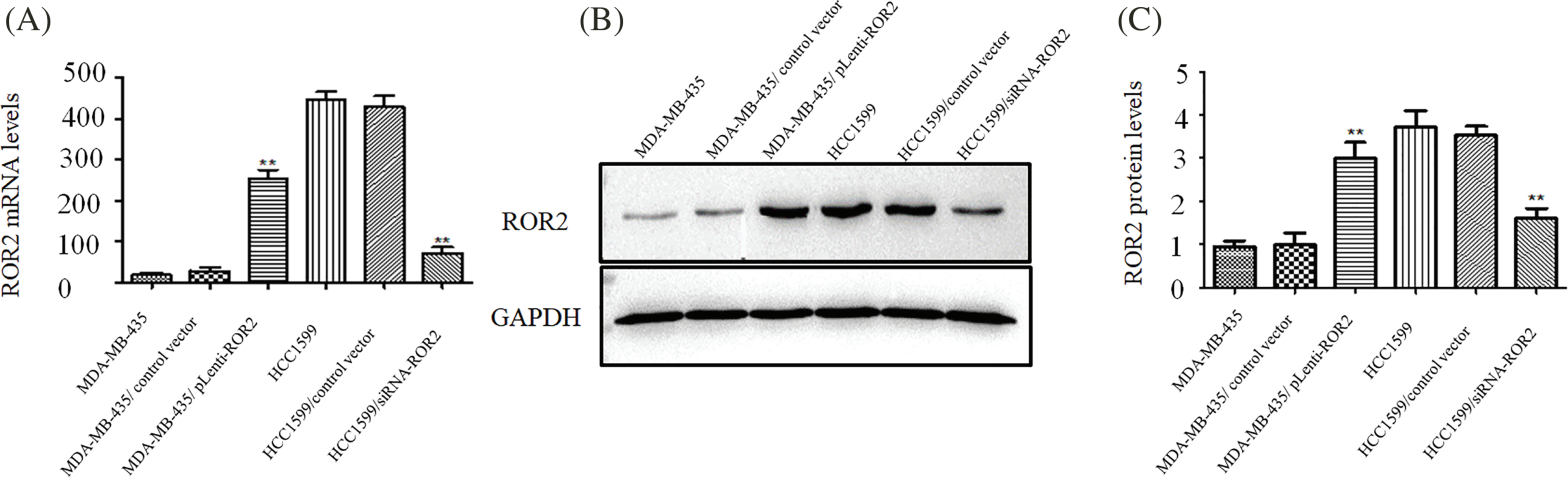
Generating HCC1599 and MDA-MB-435 cells with stable knockdown and overexpression of ROR2. HCC1599 cells were transfected with ROR2 siRNA plasmid or non-targeting control, and selected by 5 μg/ml puromycin. Stable ROR2 expression MDA-MB-435 cells were generated by transfection with pLenti-ROR2 plasmid and selected by 5 μg/ml puromycin. ROR2 expression was analyzed at mRNA (A) and protein (B and C) levels. Data are presented as mean ± SD (n = 3, **p < 0.01 *vs*. parent or vector transfected control cells).

ROR2 expression was silenced in HCC1599 cells transfected with siROR2 at mRNA (Fig. 2A) and protein level (Figs. 2B and 2C) compared to control siRNA transfected cells. After selection with puromycin, stable HCC1599/siRNA-ROR2 cells with knockdown of ROR2 were constructed. Similarly, MDA-MB-435 cells overexpressing ROR2 were constructed by transfection with pLenti-ROR2, followed by selection with puromycin. MDA-MB-435/pLenti-ROR2 cells showed increased ROR2 expression at mRNA (Fig. 2A) and protein level (Figs. 2B and 2C) compared to control vector transfected cells.

### ROR2 inhibited apoptosis and chemosensitivity of TNBC cells

**Figure 3:**
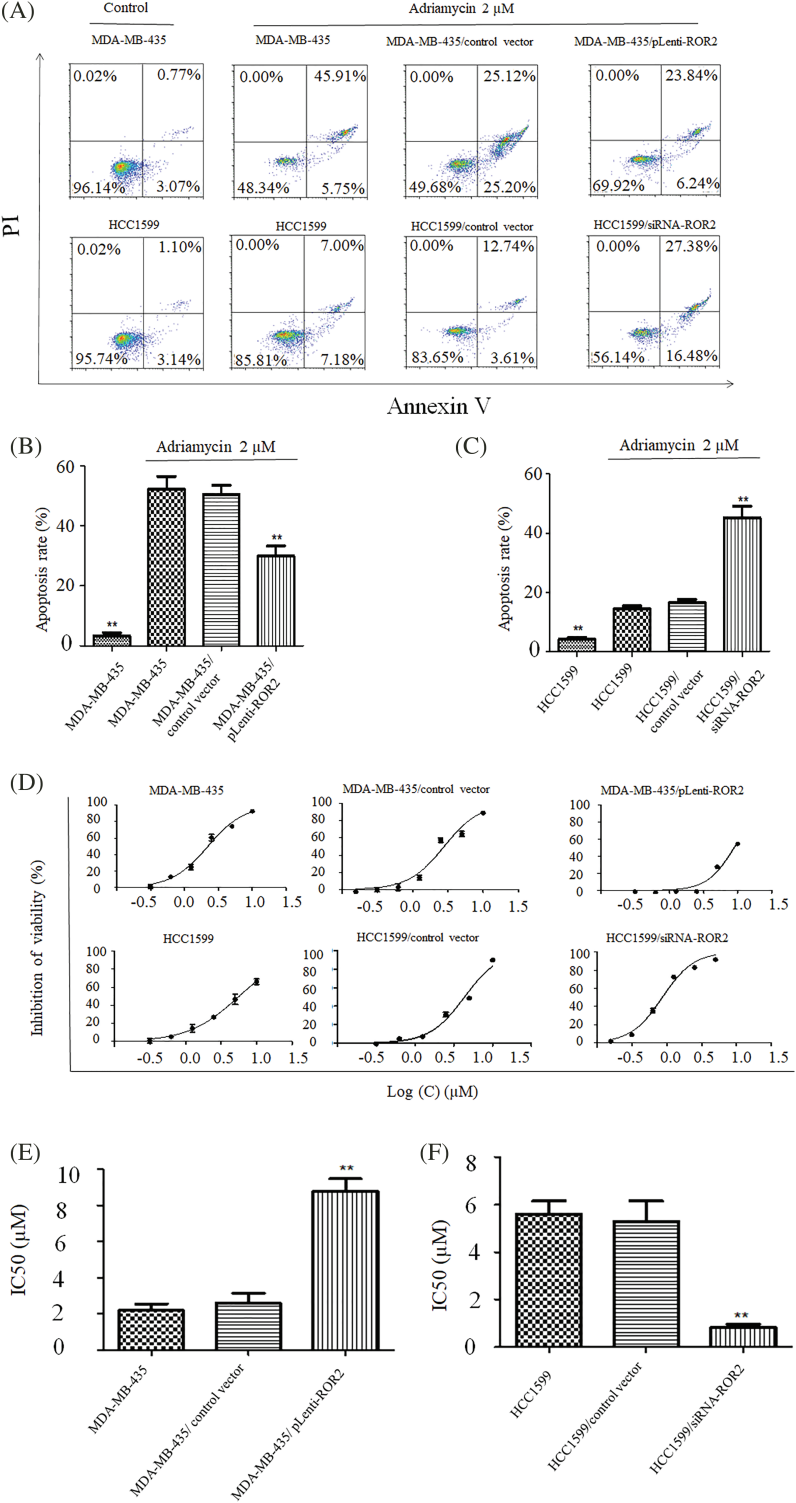
ROR2 regulated Adriamycin induced apoptosis of TNBC cells. MDA-MB-435 (A and B) and HCC1599 (A and C) cells with altered ROR2 expression were exposed to 2 uM adriamycin and apoptosis was examined by flow cytometry. ROR2 altered chemosensitivity of MDA-MB-435 (D and E) and HCC1599 (D and F). CCK-8 assay of cells treated with adriamycin for 72 h, and IC50 values were calculated (D and E). Data are presented as mean ± SD (n = 3, **p < 0.01 *vs*. parent or vector control cells).

After ROR2 knockdown in HCC1599 cells and ROR2 overexpression in MDA-MB-435 cells, adriamycin induced apoptosis in MDA-MB-435 cells was weakened in ROR2 overexpressing MDA-MB-435 cells, while ROR2 knockdown HCC1599 cells exhibited significantly higher induction of adriamycin-mediated apoptosis compared to control HCC1599 cells (Figs. 3A–3C).

CCK-8 assay showed the chemosensitivity of TNBC cells to Adriamycin. IC50 value increased from 2.20 μM in MDA-MB-435 cells to 8.84 μM in MDA-MB-435/pLenti-ROR2 cells, but decreased from 5.63 μM in HCC1599 cells to 0.85 μM in HCC1599/siRNA-ROR2 cells (Figs. 3D–3F). The changes in IC50 values indicate that chemosensitivity to adriamycin significantly increased in HCC1599 cells upon ROR2 knockdown, but decreased in MDA-MB-435 cells with ROR2 overexpression.

### ROR2 activated PI3K/AKT/mTOR signaling in TNBC cells

**Figure 4:**
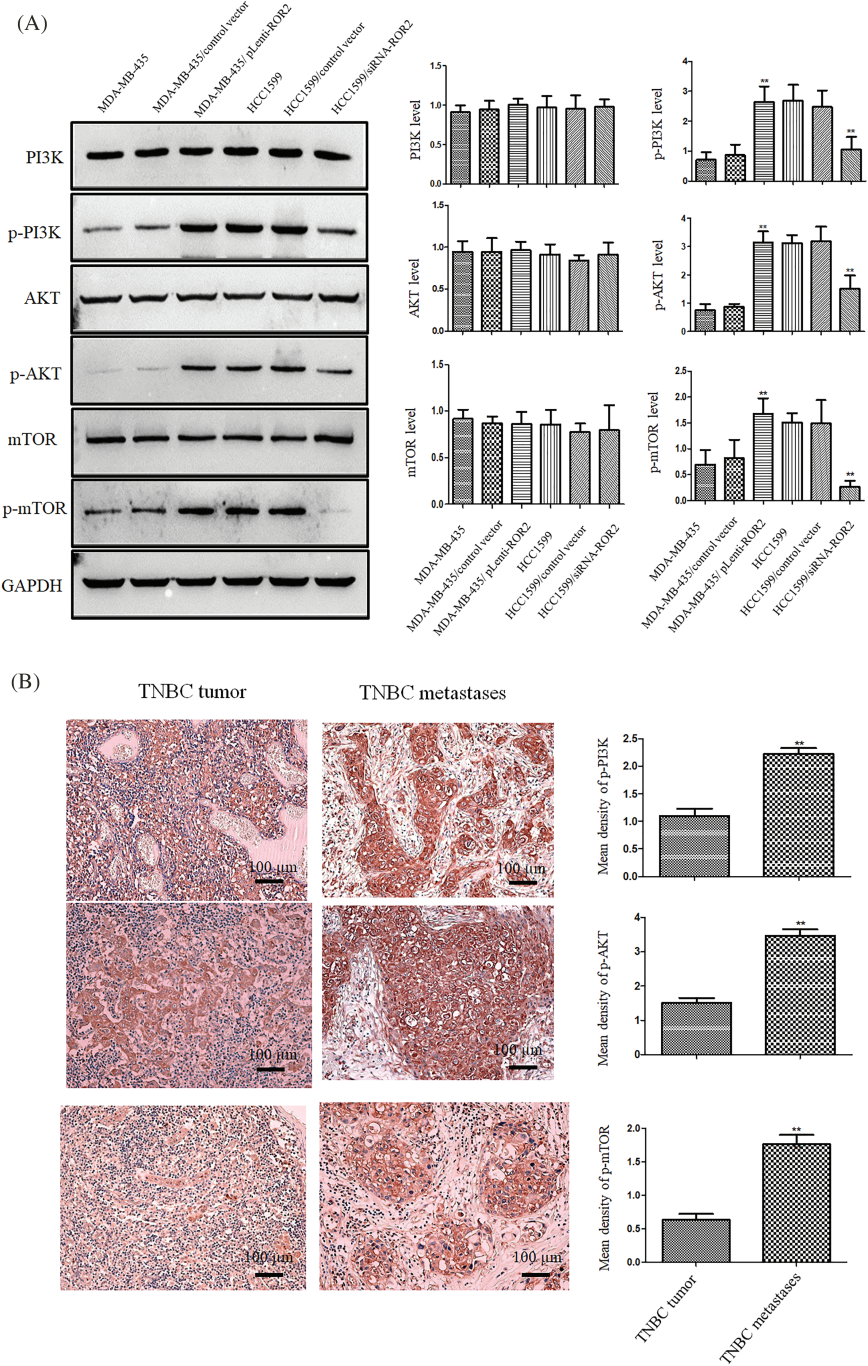
ROR2 activated PI3K/AKT/mTOR pathway in TNBC cells. (A) Western blot analysis of p-PI3K p85 (Tyr458)/p55 (Tyr199), PI3K, p-AKT (Ser 473), AKT, p-mTOR (Ser 2448) and mTOR proteins in TNBC cell lines (mean ± SD, n = 3, **p < 0.01 *vs*. parental cells). (B) IHC of p-PI3K, p-AKT, and p-mTOR in primary and metastatic TNBC tissues (n = 20). Scale bar: 100 μm. Data are presented as mean ± SD. **p < 0.01. *vs*. TT. TT: TNBC tumor, TM: TNBC metastases.

Western blot analysis showed that the levels of p-PI3K, p-AKT and p-mTOR increased in ROR2 overexpressing MDA-MB-435 cells. However, ROR2 knockdown in HCC1599 cells reduced the levels of p-PI3K, p-AKT, and p-mTOR with no significant changes in PI3K, AKT, and mTOR levels (Fig. 4A). Furthermore, p-AKT, p-PI3K and p-mTOR levels were significantly higher in tissues from metastatic TNBC compared to those in tissues from primary TNBC cancer (Fig. 4B).

### ROR2 promoted TNBC cell migration and invasion

**Figure 5:**
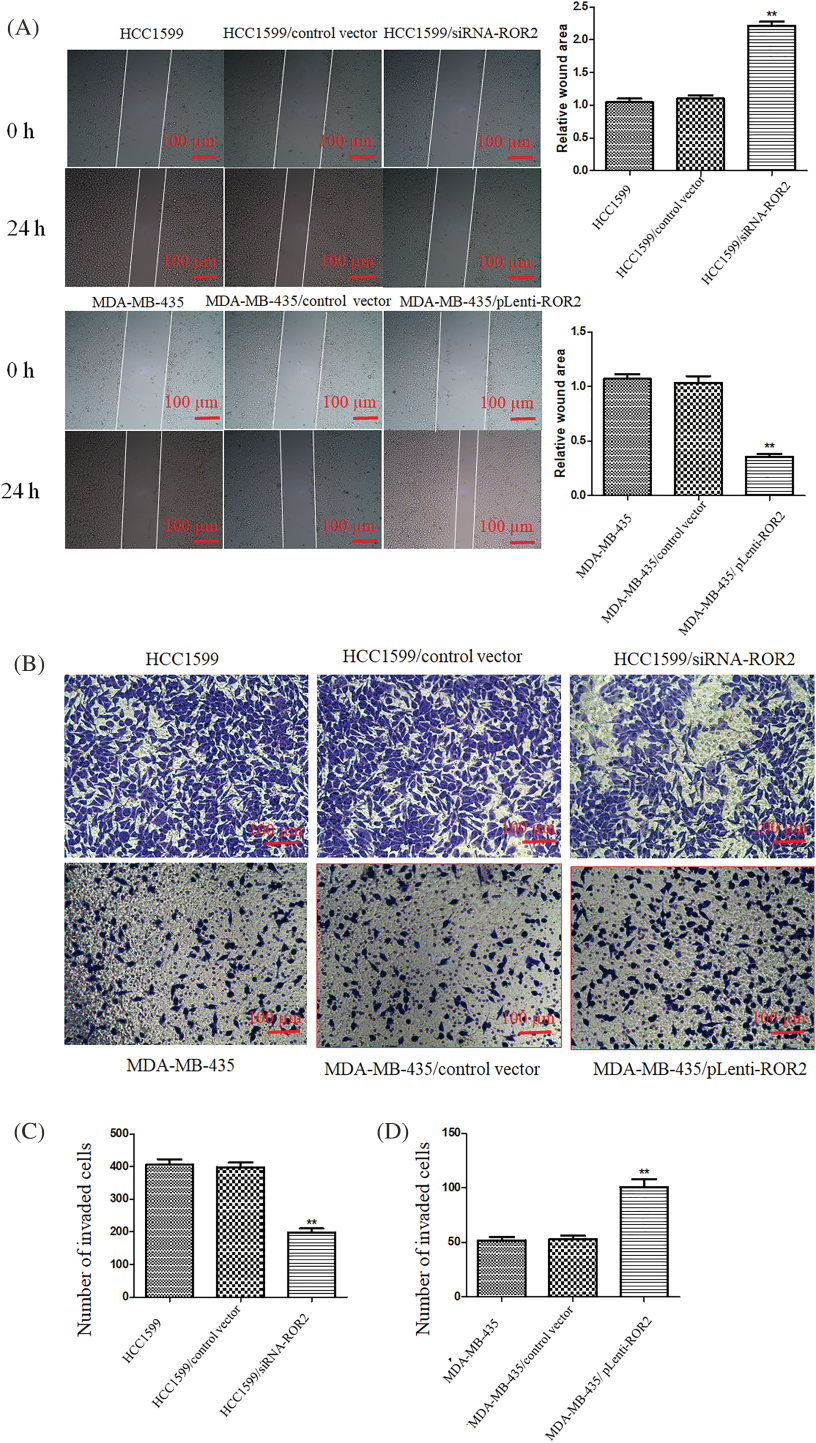
ROR2 promoted migration and invasion potential of TNBC cells. (A) Representative images (Left panel) showing wound closure after 24 h in TNBC cells. magnification 200×. Right panel: The quantification of wound area. Cells that invaded to the lower side of the chamber were counted (B). Transwell invasion assay. (C and D) The quantification of invaded cells. Scale bar: 100 μm. Data are presented as mean ± SD (n = 5, **p < 0.01 *vs*. controls).

Wound healing assay and Matrigel assay of ROR2 overexpressing MDA-MB-435 cells or ROR2 depleted HCC1599 cells showed that migration and invasion potential significantly decreased in HCC1599/siRNA-ROR2 cells but significantly elevated in MDA-MB-435/pLenti-ROR2 cells (Figs. 5A and 5B).

**Figure 6:**
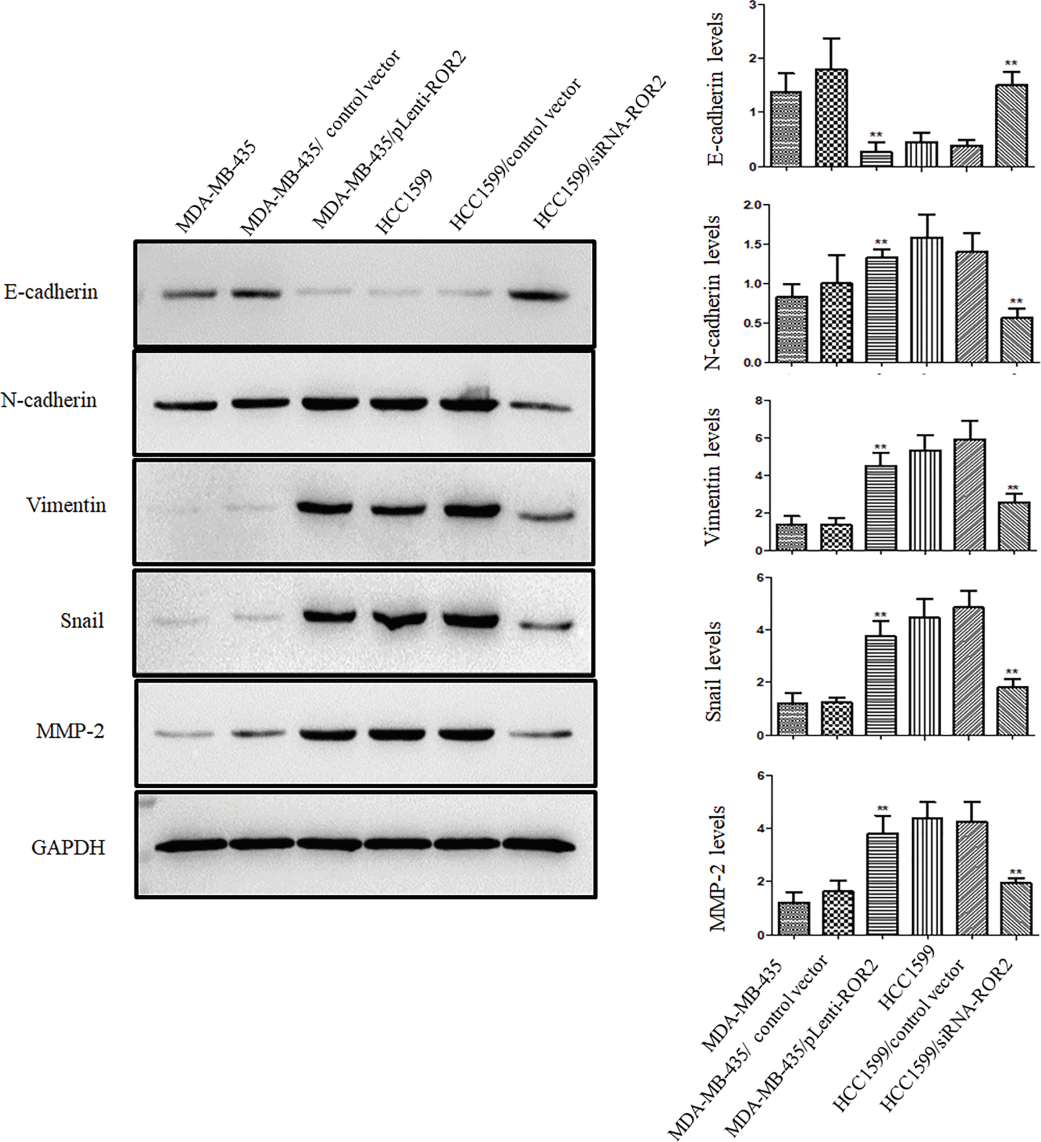
ROR2 expression correlated positively with metastatic phenotype of TNBC cells. Representative blots (Left panel) and histograms (Right panel) of E-cadherin, N-cadherin, vimentin, Snail and MMP-2 proteins in MDA-MB-435/pLenti-ROR2, HCC1599/siRNA-ROR2, and their respective parental cells. Data are presented as mean ± SD (n = 3, **p < 0.01 *vs*. parental cells).

Furthermore, ROR2 overexpressing cells showed the upregulation of N-cadherin, vimentin, MMP-2 and Snail, and the downregulation of E-cadherin, compared to MDA-MB-435 parental cells. The expression of these proteins in HCC1599/siRNA-ROR2 cells showed the opposite trend compared to HCC1599 parental cells (Fig. 6).

### ROR2 inhibited apoptosis and chemosensitivity in adriamycin-resistant MDA-MB-435 cells

**Figure 7:**
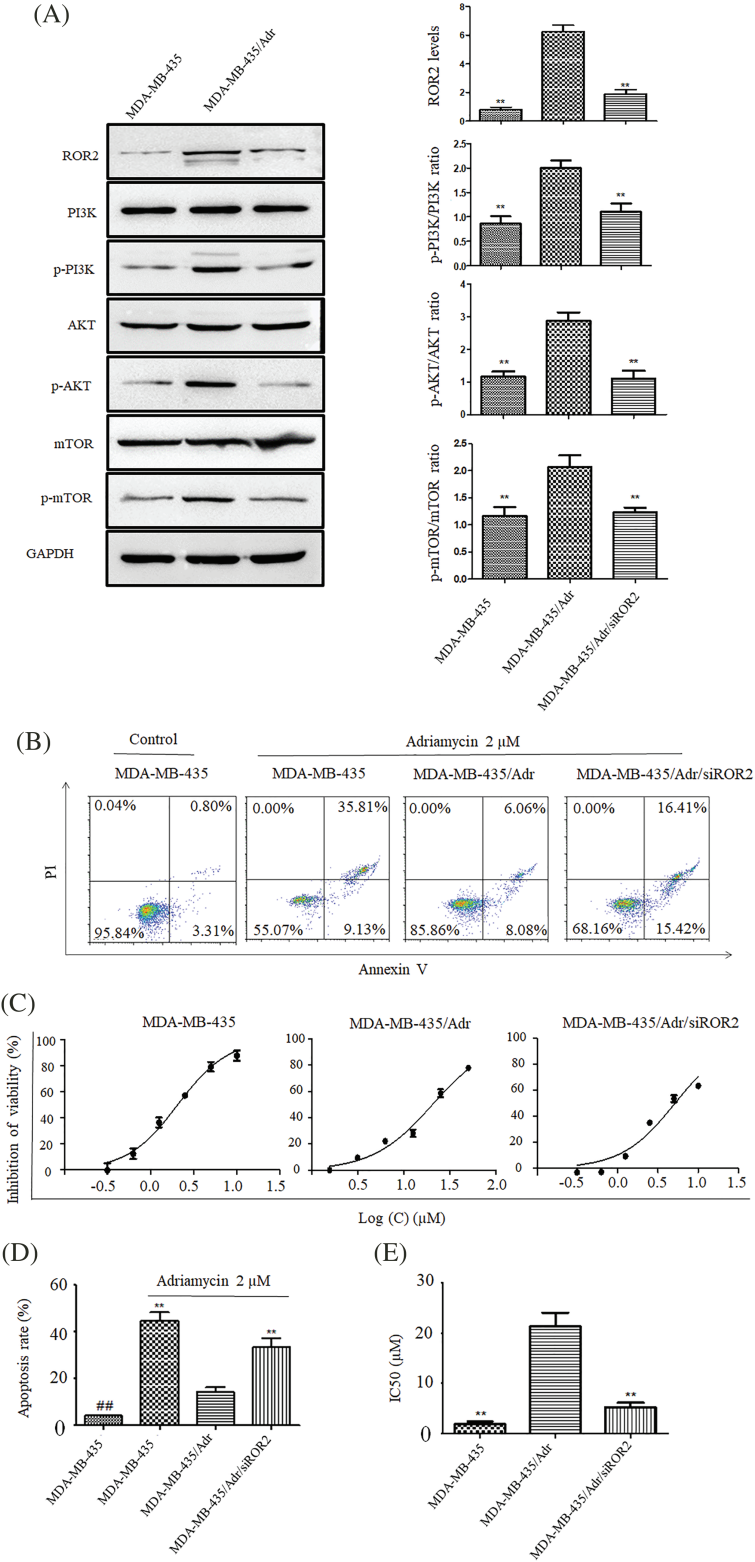
ROR2 affects PI3K/AKT/mTOR signaling in MDA-MB-435/Adr cells. (A) Western blot analysis of ROR2, p-PI3K, PI3K, p-AKT, AKT, p-mTOR and mTOR proteins in MDA-MB-435/Adr cells. Data present as mean ± SD. n = 3, **p < 0.01 *vs*. parental cells. (B and D) Apoptosis of MDA-MB-435 and MDA-MB-435/Adr cells exposed to 2 uM adriamycin for 24 h. Data present as mean ± SD (n = 3, **p < 0.01). (C and E) CCK-8 assay of MDA-MB-435 and MDA-MB-435/Adr cells exposed to 2 uM adriamycin for 72 h, and IC50 values were calculated. Data are presented as mean ± SD (n = 3, **p < 0.01 *vs*. MDA-MB-435/Adr group. ##*p* < 0.01 *vs*. MDA-MB-435 adriamycin treated group).

Using adriamycin-resistant MDA-MB-435/Adr cells, we investigated the role of ROR2 in drug resistance of TNBC. Western blot analysis showed upregulated ROR2 expression in MDA-MB-435/Adr cells compared to MDA-MB-435 cells. After knockdown of ROR2, p-PI3K, p-AKT and p-mTOR levels significantly decreased in MDA-MB-435/Adr cells with no changes in total PI3K, AKT and mTOR levels (Fig. 7A). While adriamycin induced apoptosis in MDA-MB-435 cells, the effect was weakened in MDA-MB-435/Adr cells. Moreover, ROR2 knockdown in MDA-MB-435/Adr led to increased apoptosis rate. CCK-8 assay showed that IC50 values of adriamycin increased from 2.05 μM in MDA-MB-435 cells to 20.35 μM in MDA-MB-435/Adr cells, and to 5.15 μM in MDA-MB-435/Adr/siROR2 cells (Figs. 7B–7E).

### ROR2 promotes migration and invasion of chemoresistant MDA-MB-435/Adr cells

**Figure 8:**
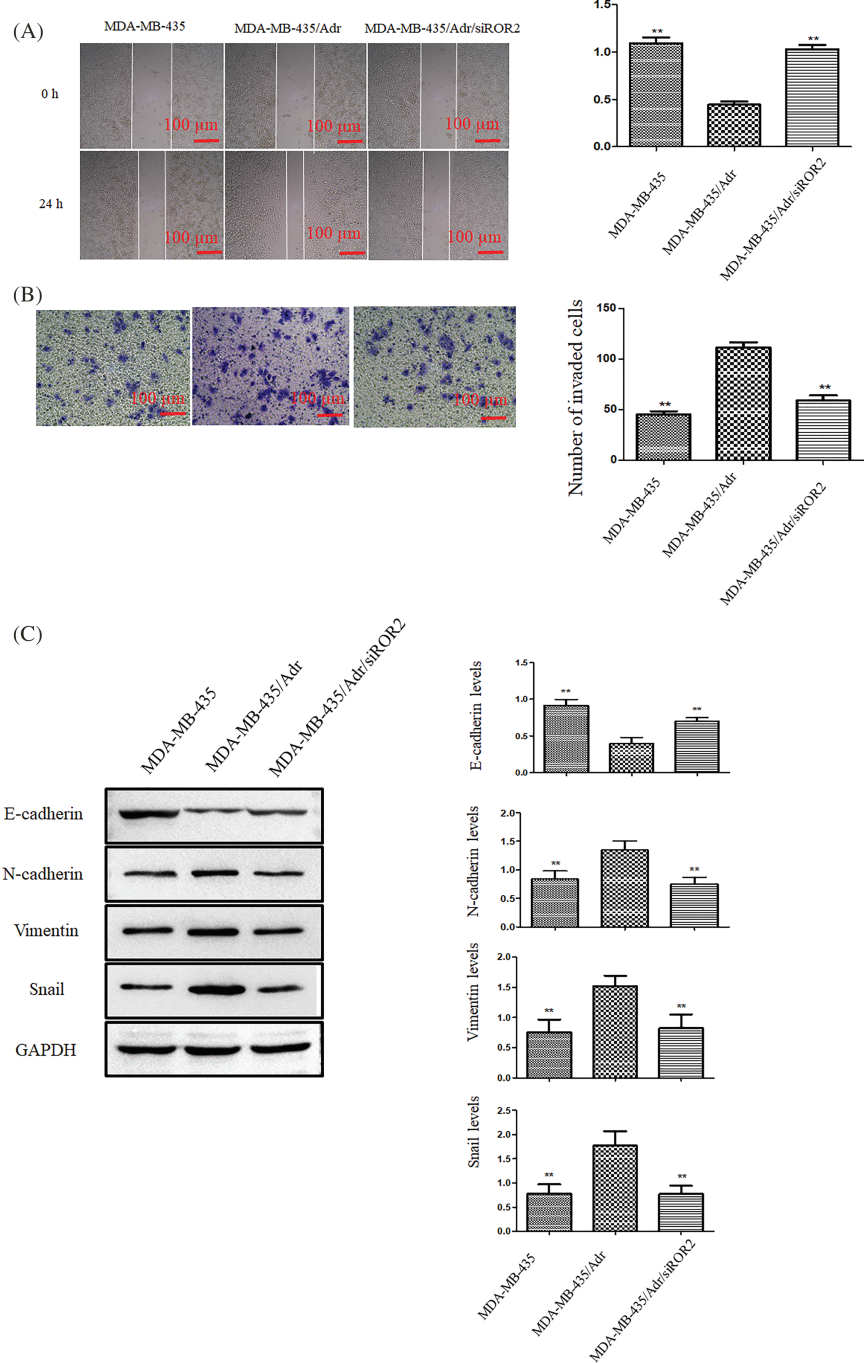
ROR2 promoted migration and invasion of MDA-MB-435/Adr cells. (A) Representative images (Left panel) showing wound closure after 24 h. The quantification of wound area (Right panel). (B) Transwell invasion assay. Scale bar: 100 μm. (C) The analysis of E-cadherin, N-cadherin, vimentin, Snail, and MMP-2 protein in MDA-MB-435, MDA-MB-435/Adr and MDA-MB-435/Adr/siROR2 cells. Data are presented as mean ± SD (n = 3, **p < 0.01 *vs*. MDA-MB-435/Adr cells).

The migration and invasion potential significantly elevated in MDA-MB-435/Adr cells and significantly weakened in MDA-MB-435/Adr/siROR2 cells (Figs. 8A and 8B). Furthermore, Western blot analysis demonstrated significant upregulation of N-cadherin, vimentin, Snail and MMP-2, along with downregulation of E-cadherin in MDA-MB-435/Adr cells. However, these proteins showed opposite trend in MDA-MB-435/Adr/siROR2 cells compared to MDA-MB-435/Adr parental cells (Fig. 8C).

## Discussion

ROR2 has been proposed as an oncogene [13,14]. Moreover, ROR2 expression was increased in NSCLC [15]. In this study, we showed that higher ROR2 expression in TNBC tissues correlated with metastatic phenotype in TNBC, indicating that ROR2 may promote TNBC metastasis.

PI3K/AK/mTOR pathway plays an essential role in tumor metastasis [16]. ROR2 overexpression promoted renal cancer by regulating PI3K/AKT signaling [17]. In this study, knockdown of ROR2 inhibited TNBC cell migration and invasion. Furthermore, our study showed that ROR2 may activate PI3K/AKT/mTOR signaling to regulate metastasis of TNBC.

PI3K/AKT/mTOR pathway is often deregulated in different cancers [18–21]. Previous studies have shown that certain small molecule compounds exert inhibitory effects on breast cancer by inhibiting PI3K/AKT signaling [22]. Rehman et al. demonstrated that downregulation of ROR2 inhibited thyroid cancer cell proliferation and invasion via PI3K/AKT signaling [23]. Here, we showed that ROR2 knockdown inhibited TNBC cell migration and invasion and increased chemosensitivity *in vitro*.

Recognized as a noncanonical Wnt receptor, ROR2 regulates a variety of cell activities such as breast cancer cell invasion [24]. Regulation of ROR2 by siRNA mediated knockdown may inhibit all downstream signaling pathways, including but not limited to PI3K/AKT/mTOR signaling. In contrast, small molecule compounds of PI3K/AKT signaling inhibitors are specific and exclusive.

This study has limitations. The sample size is relatively small and studies with large sample size are necessary to confirm high expression of ROR2 in metastatic TNBC tissues.

In conclusion, for the first time we demonstrate that ROR2 and PI3K/AKT/mTOR signaling regulate drug resistance and metastasis in TNBC. ROR2 may be a novel target to limit TNBC invasion and metastasis.

## Data Availability

All data are available upon request to correspondence author.
